# Deciphering CHFR Role in Pancreatic Ductal Adenocarcinoma

**DOI:** 10.3389/fmed.2021.720128

**Published:** 2021-11-19

**Authors:** Iranzu González-Borja, Emilia Alors-Pérez, Irene Amat, Laura Alonso, Cristina Viyuela-García, Saioa Goñi, José C. Reyes, María Ceballos-Chávez, Irene Hernández-García, Marina E. Sánchez-Frías, Enrique Santamaría, Socorro Razquin, Álvaro Arjona-Sánchez, Virginia Arrazubi, Jairo Pérez-Sanz, Ruth Vera, Joaquín Fernández-Irigoyen, Justo P. Castaño, Antonio Viúdez

**Affiliations:** ^1^OncobionaTras Lab, Navarrabiomed, Complejo Hospitalario de Navarra, Universidad Pública de Navarra, Instituto de Investigación Sanitaria de Navarra, Pamplona, Spain; ^2^Hormones and Cancer Group, Maimonides Institute for Biomedical Research of Cordoba (IMIBIC), Córdoba, Spain; ^3^Department of Cell Biology, Physiology, and Immunology, University of Córdoba, Córdoba, Spain; ^4^Reina Sofia University Hospital, Córdoba, Spain; ^5^Centro de Investigación Biomédica en Red (CIBER) Fisiopatología de la Obesidad y Nutrición, Córdoba, Spain; ^6^Pathology Department, Complejo Hospitalario de Navarra, Pamplona, Spain; ^7^Surgery Service, Reina Sofia University Hospital, Córdoba, Spain; ^8^Centro Andaluz de Biología Molecular y Medicina Regenerativa, Consejo Superior de Investigaciones Científicas-Universidad de Sevilla-Universidad Pablo de Olavide, Seville, Spain; ^9^Medical Oncology Department, Complejo Hospitalario de Navarra, Pamplona, Spain; ^10^Pathology Service, Reina Sofia University Hospital, Córdoba, Spain; ^11^Proteomics Platform, Clinical Neuroproteomics Unit, Navarrabiomed, Complejo Hospitalario de Navarra, Universidad Pública de Navarra, Instituto de Investigación Sanitaria de Navarra, Pamplona, Spain; ^12^Medical Affairs Services, ICON plc, North Wales, PA, United States

**Keywords:** pancreatic ductal adenocarcinoma (PDAC), DNA methylation, checkpoint with forkhead and ring finger domains (*CHFR*), methylation, immunohistochemistry (IHC)

## Abstract

Checkpoint with forkhead-associated and ring finger domains (*CHFR*) has been proposed as a predictive and prognosis biomarker for different tumor types, but its role in pancreatic ductal adenocarcinoma (PDAC) remains unknown. The aim of this study was two-pronged: to review the role of *CHFR* in PDAC and evaluating *CHFR* as a potential predictive biomarker in this disease. For this purpose, we first explored the CHFR messenger (m)RNA expression and promoter methylation through the TCGA database. Secondly, the CHFR expression and promoter methylation were prospectively evaluated in a cohort of patients diagnosed with borderline (*n* = 19) or resectable (*n* = 16) PDAC by immunohistochemistry (IHC), methylation specific-PCR (MSP), and pyrosequencing. The results from the TCGA database showed significant differences in terms of progression-free survival (PFS) and overall survival (OS) based on the CHFR mRNA expression, which was likely independent from the promoter methylation. Importantly, our results showed that in primarily resected patients and also the entire cohort, a higher CHFR expression as indicated by the higher IHC staining intensity might identify patients with longer disease-free survival (DFS) and OS, respectively. Similarly, in the same cohorts, patients with lower methylation levels by pyrosequencing showed significantly longer OS than patients without this pattern. Both, the CHFR expression intensity and its promoter methylation were established as independent prognostic factors for PFS and OS in the entire cohort. In contrast, no significant differences were found between different methylation patterns for *CHFR* and the response to taxane-based neoadjuvant treatment. These results suggest the potential role of the higher expression of CHFR and the methylation pattern of its promoter as potential prognostic biomarkers in PDAC, thus warranting further comprehensive studies to extend and confirm our preliminary findings.

## Introduction

Pancreatic ductal adenocarcinoma represents the seventh leading cause of cancer-related deaths worldwide (1, 2), remaining as one of the most lethal types of cancers despite the efforts to improve its diagnosis, surgical procedures, and treatments. In 2020, 495,773 cases of pancreatic ductal adenocarcinoma (PDAC) and 466,003 deaths due to this fatal disease were estimated by the global cancer incidence, mortality, and prevalence (GLOBOCAN) (3). The incidence rates of PDAC are increasing around 3% yearly, regardless of gender, and being most frequently diagnosed in the elderly population between 65 and 74 years old (4). The cancer statistics for 2021 shows that PDAC will be the fourth cause of cancer-related deaths in the United States (USA) with 60,430 estimated cases and 48,220 estimated deaths. The 5-year overall survival rate (5yOS) for PDAC remains the lowest (10%) in comparison with other cancers (5). Furthermore, a recent study reveals that PDAC is expected to become the second leading cause of cancer-related deaths, after lung cancer, by 2030 in the USA (6). Thus, it is clear that novel tools to improve PDAC diagnosis and treatment are urgently required.

The checkpoint with forkhead-associated and ring finger domains (CHRF) gene encodes a protein ubiquitously expressed in normal human tissues implicated in a checkpoint regulating the entry into mitosis (7, 8). It is well-known that checkpoint regulators are highly important to prevent the propagation of cells with damaged genomes, which if allowed, could lead to a higher risk of developing cancer (9). In situations wherein microtubule-targeting drugs are used, the main role of CHFR is to delay cell entry into the metaphase (10), which does not happen under homeostatic conditions (8). Structurally, CHFR is a 664 amino acids nuclear protein that has different functional domains: (1) an N-terminal forkhead-associated (FHA) domain that checks the phosphorylation status of specific threonine residues of target proteins (11); (2) a central RING-finger (RING) domain with ubiquitination activity that is needed for its checkpoint function in the G2/M transition; (3) a C-terminal cysteine-rich region also needed to bind Aurora A (12)- one of the key kinases that regulate mitotic events including the entry into mitosis and plays a critical role in carcinogenesis- (13, 14); and (4) the poly (ADP-ribose)-binding zinc finger (PBZ) motif (8) that is required for blocking the transition into mitosis in the presence of microtubule drugs (15). In different ways, CHFR can delay the mitotic entry into metaphase. Thus, CHFR induces the ubiquitination of Polo-like kinase 1 (Plk1) resulting in a delay in mitotic entry (16). Aurora A interacts with Plk1 inducing its phosphorylation and thus leading to its activation in the G2. Aurora A is also dependent on Plk1 to localize to the centrosomes in the late G2, wherein it recruits an important mitotic cyclin, cyclin B1, to the centrosomes, and phosphorylates Cdc25B which then triggers the activation of the cyclin, B1-Cdk1 complex. Thanks to these previous mechanisms, Aurora and Plk1 contribute to the proper initiation of mitosis. As mentioned earlier, CHFR can also bind Aurora A, leading to its destabilization (17). Moreover, some studies have shown an inverse correlation between the loss of CHFR function and Aurora A levels in CHFR-/- mouse embryonic fibroblasts (12), in prostate and breast cancer cell lines models (18), and also in colorectal cancer tissue samples (19).

The promoter methylation of *CHFR* has been described in several tumor types, such as gastric and colorectal adenocarcinoma (20–23), and has been also described as a potential biomarker for taxanes (24), irinotecan (25), or poly (ADP-ribose) polymerase (PARP) inhibitors (26) sensitivity. *In vitro* and non-clinical studies showed an increase in taxanes sensitivity when the suppression of CHFR expression (27) or *CHFR* methylation was generated (28). These data are also supported by different studies that reported how the *CHFR* promoter methylation predicted the response to taxanes in metastatic non-small-cell lung carcinoma (NSCLC) (24) or gastric adenocarcinoma (22), similar to what has been shown in patients with colorectal adenocarcinoma treated with irinotecan in which a significantly longer time-to-progression (TTP) was observed in those with methylated *CHFR*. Although some data support the association between *CHFR* promoter methylation and better outcomes (29), other studies have correlated *CHFR* methylation with worse progression-free survival (PFS) and overall survival (OS), having even been proposed as an independent predictor of recurrence in colorectal adenocarcinoma (30). In contrast with a latter report, other studies on NSCLC reported worse OS when a high expression of CHFR was detected, questioning the role of CHFR as a hypothetical suppressor gene (31). In this respect, some studies indicate a strong positive correlation between *CHFR* promoter methylation and the risk of developing gastric cancer (32), *CHFR* promoter methylation is higher in tumors than in normal gastric tissues and is significantly associated with positive node metastasis (33), suggesting that although *CHFR* promoter hypermethylation seemed to be associated with gastric neoplasia, it may also play a protective role during the carcinogenesis process.

To date, only three studies have described the role of CHFR in PDAC. The first one, published in 2017, described retrospectively, by immunohistochemistry (IHC), the expression of CHFR in both the pancreatic cell lines and PDAC tissue samples from a cohort of resected patients without neoadjuvant treatment (34), showing that higher CHFR expression was associated with earlier T-stage (pT1-2), but not with significant OS differences. Recently, the first article that showed methylation data in healthy, adjacent, and tumoral tissue regarding *CHFR* did not find significant differences between them (35). Despite this, they found that *CHFR* was methylated in 31% (12/38) of the cases in the tumoral tissue, and reported a correlation between *CHFR* methylation and the presence of node metastasis. Nevertheless, the results did not show significant OS differences depending on the *CHFR* methylation pattern (*p* = 0.698). Finally, Wu and colleagues established a genomic-clinical nomogram with five genes (including *CHFR*) which could serve as a prognostic biomarker in resectable PDAC (36), showing that it could be an effective tool in this scenario.

Undoubtedly, all these studies provide evidence that further analyses are needed to describe the relevance of CHFR in PDAC. In this sense, the main aim of our study is to contribute to the knowledge about the role of CHFR in this devastating disease through a prospective characterization of the expression of the CHFR protein and by quantifying its promoter methylation percentage. Subsequently, we correlate these results with clinical and histopathological parameters in patients diagnosed with PDAC who were treated with irinotecan or taxanes-based regimens before and/or after the planned surgery.

## Materials and Methods

### Bioinformatic Analysis Using cBioPortal

The cBio Cancer Genomics Portal (http://cbioportal.org) is an open-access resource for cancer genome data that provides access to the data of more than 5,000 tumor samples from 20 different cancer studies (37), including all data from The Cancer Genome Atlas (TCGA) (38). Among the available pancreatic adenocarcinoma studies in the cBioPortal, we analyzed the data from 186 patients in the cohort available as “TGCA, PanCancer Atlas.” The CHFR mRNA expression data and clinical information were extracted from cBioPortal and the *CHFR* methylation data was downloaded from www.firebrowse.org (Supplementary Figures 1, 2).

### Patients and Tumor Samples

The Ethics Committee of the Government of Navarre approved the present prospective project. Informed consent was obtained from all the subjects, with all procedures performed in accordance with the World Medical Association (WMA) Declaration of Helsinki. Those patients diagnosed and considered with potentially resectable (borderline) or initially resectable PDAC were included from two Spanish reference centers (Complejo Hospitalario of Navarre, Pamplona, and Reina Sofia University Hospital, Córdoba). Specialized pathologists in both sites selected formalin-fixed paraffin-embedded (FFPE) tissue samples and several sections were used for methylation analysis and IHC. The clinical and demographic data of patients are shown in Table 1. The demographic clinical and pathological variables were extracted from the electronic clinical system including age, gender, date of diagnosis, date of surgery, treatments received, response to treatment, date of progression, and date of death (among others).

**Table 1:** Clinical and demographic data of patients.

**Variable**	**Borderline patients** **(*N* = 19) (%)**	**Resectable patients** **(*N* = 16) (%)**	**Chi-Square test**
Median age	63 (41–81)	66 (50–80)	*p* = 0.569
Gender			*p* = 0.052
Female	8 (42.1)	12 (75)	
Male	11 (57.9)	4 (25)	
Neoadjuvant therapy			
Nab-paclitaxel-gemcitabine	14 (73.7)		
Folfirinox	5 (26.3)		
Neoadjuvant ChemoRT			
Yes	4 (21.1)		
No	15 (78.9)		
Neoadjuvant SBRT	3 (15.8)		
Response			
Partial response	10 (52.6)		
Stable disease	9 (47.4)		
Surgery	All	All	
Pathologic response			
1	4 (21.1)		
2	10 (52.6)		
3	5 (26.3)		
Perineural invasion			*p* = 0.187
Yes	14 (73.7)	15 (93.8)	
No	5 (26.3)	1 (6.3)	
Vascular invasion			*p* = 0.738
Yes	9 (47.4)	9 (56.3)	
No	10 (52.6)	7 (43.8)	
R			*p* = 0.244
R0	13 (68.4)	14 (87.5)	
R1	6 (31.6)	2 (12.5)	
Lymph nodes involved			*p* = 0.493
Yes	12 (63.2)	12 (75)	
No	7 (36.8)	4 (25)	
pT			
1	9 (47.4)	2 (12.5)	*p* = 0.006**
2	7 (36.8)	4 (25)	
3	3 (15.8)	9 (56)	
4	0	1 (6.3)	
pN			*p* = 0.415
0	7 (36.8)	4 (25)	
1	7 (36.8)	10 (62.5)	
2	5 (26.3)	2 (12.5)	
Adjuvant treatment			*p* = 0.018*
Yes	11 (57.9)	15 (93.8)	
No	8 (42.1)	1 (6.3)	
Adj chemoRT			*p* = 0.758
Yes	1 (5.3)	1 (6.3)	
No	15 (78.9)	15 (93.8)	

*CHEMORT, Concomitant Chemotherapy and Radiotherapy; SBRT, Stereotactic Body Radiation Therapy; R, Residual tumor; R0, no cancer cells seen microscopically at the primary tumor site; R1, cancer cells present microscopically at the primary tumor site; pT, pathological tumor size AJCC 8th edition; pN, pathological lymph node staging AJCC 8th edition*.

### Cell Lines

The human pancreatic adenocarcinoma and colorectal cell lines were cultured in a humidified atmosphere containing 5% carbon dioxide (CO2) at 37°C temperature. AsPC-1 (ATCC: CRL-162), BxPC-3 (ATCC: CRL-1687), MIA PaCa-2 (ATCC: CRL-1420), NP-18 (kindly provided by Dr. Arasanz from Navarrabiomed Biomedical Research Center, Pamplona, Spain) and PANC-1 (ATCC: CRL-1469), LOVO (ATCC: CCL-229), HT-29 (ATCC: HTB-38), HCT116 (ATCC: CCL-247), SW480 (ATCC: CCL-228) and DLD-1 (ATCC: CCL-221) were grown in Dulbecco's Modified Eagle Medium (DMEM) or Roswell Park Memorial Institute (RPMI) 1640 supplemented with 10% fetal bovine serum (FBS), 1% antibiotics (penicillin (100 U/ml), and streptomycin (100 μg/ml) at 50–80% confluence. All these cell lines were adherent with epithelial morphology and presented a high proliferative rate.

### Detection of *CHFR* Promoter Methylation

The *CHFR* promoter methylation status was evaluated by using the Methylation specific PCR (MS-PCR) technique and pyrosequencing assay for the quantitative methylation analysis. These methods distinguish between unmethylated and methylated cytosines on the DNA sequence after the bisulfite treatment of DNA that converts unmethylated cytosines into uracil and subsequently to thymine during PCR. The DNA from the colorectal cell lines was assessed to validate the performance of the assay (Supplementary Figure 3).

### DNA Extraction and Bisulfite Conversion

The DNA extraction and bisulfite treatment of the FFPE slides of the patients were performed with an EpiTect® Fast FFPE Bisulfite Kit (Catalog 59844 QIAGEN, Hilden, Germany), following the recommendations of the manufacturer.

The DNA extraction from the cell lines was different. The cell lines were amplified, and the cellular pellet was obtained by scraping the cells on ice-cold phosphate-buffered saline (PBS), then the pellet was centrifuged to remove the PBS and stored at −80°C. For the DNA extraction, 400 μl of proteinase K buffer [10 mM TRIS pH = 8, 50 mM sodium chloride (NaCl), 25 mM EDTA pH8, 0.5% SDS] and 10 μl of proteinase K (stock 10 mg/ml) were added to each sample and incubated overnight at 55°C with stirring. Then, 200 μl of NaCl 6M were added, mixed briefly, and centrifuged at 12,000 g 30 min at room temperature (RT). The supernatant was transferred into a new tube and 900 μl of ethanol 100% was added for DNA precipitation. The DNA was washed twice with 1 ml of ethanol 70% and centrifuged at 4,000 g 2 min at RT. The DNA was dried and re-suspended in 50 μl of H_2_O.

### MS-PCR

The *CHFR* promoter methylation status was determined by using two sets of primers as previously described by Pelosof (24) (Supplementary Table 1). The PCR was performed in a 25 μl final reaction volume containing 0.25 μl of JumpStart^TM^ Tac Polymerase and 2.5 μl of 10X Tac Buffer (D9307, SIGMA-ALDRICH), 0.3 μl of each primer (50 μM), dNTPs 1.25 μl (10 mM) (BIO39028, BIOLINE, London, UK), H2O 14.4 and 2 μl of the sample, with 59°C annealing temperature and for 35 cycles.

### Pyrosequencing

In the pyrosequencing assay, the *CHFR* region analyzed was ACATGGCGCCGACCGCAGCCACTTCCGTGATCCGCAGGCGA, which contains 6 CpG sites (Catalog no. 978703 Qiagen). The *CHFR* primer sequences for the PCR amplification and sequencing steps were designed by Qiagen (Supplementary Table 1).

The amplification using *CHFR* primers was performed at an annealing temperature of 58.5°C for 45 cycles and conditions following the instructions of Qiagen. The obtained amplicon was then resolved by electrophoresis using 3% agarose gel in a 1x Tris-Acetate-EDTA Buffer in the presence of SYBR Red Safe (Catalog no. 211141, Ecogen, USA) and visualized in the UV transilluminator ChemiDoc XRS (Bio-Rad Laboratories, Hercules, CA, USA). For the pyrosequencing assay (Catalog no. 978746 Qiagen) 20 μl of the PCR products were immobilized with Streptavidin Sepharose High Performance (Ref 17-5113-01, GE Healthcare Bio-Sciences, Uppsala, Sweden) by mixing thoroughly for 10 min and then washed in the Vacuum Prep Workstation obtaining a single strand of DNA. This was followed by the annealing of the sequencing primer at 80°C for 2 min, and pyrosequencing in the PyroMark Q96 (Qiagen), using PyroMark Gold Q96 reagents. The sequencing primer is not shown because it is designed by Quiagen. Results were interpreted with the PyroMark software (Qiagen).

### CHFR IHC

The CHFR protein IHC was performed in 3 μm FFPE tumor slides using the antibody WH0055743M1, Clone 1H3-A12 from Sigma. Briefly, the deparaffinization of the slides was performed on a stove overnight at 65°C, followed by rehydration in xylene and decreasing concentrations of alcohol (100, 95, 70, and 50%), finished in water. The slides were then washed with PBS (5 min), PBS + 0.5% triton (5 min), and PBS (5 min). Heat-induced epitope retrieval (HIER) the slides were washed again in PBS (5 min), PBS + 0.5% triton (5 min), and PBS (5 min). Endogenous peroxidase was blocked with H_2_O_2_ 3% in a humidity chamber for 10 min and washed with PBS for 5 min. The slides were blocked with BSA 5% in PBS + 0.2% triton for 1 h, at room temperature in a humidity chamber. The primary antibody incubation was done overnight, at 4°C using a dilution of 1/25 of the antibody in 1% BSA in PBS + 0.2% triton. After the primary antibody incubation, slides were washed with PBS (5 min), PBS + 0.2% triton (5 min), and PBS (5 min). After that, the slides were incubated with mouse secondary antibody for 1 h at room temperature, at a concentration of 1/2,000 diluted in BSA 1% PBS + 0.2% triton. The slides were washed with PBS 10 min, PBS + 0.2% triton at 10 min, and PBS at 10 min. Chromogenic detection was performed under the microscope using 3,3'-Diaminobenzidine (DAB). The slides were counterstained with Haematoxylin-Eosin and dehydrated through increasing concentrations of alcohol and a final incubation in xylene. Finally, the slides were mounted.

The first analysis was semi-quantitative, the intensity of the stain was evaluated from 0 (no stain) to + 3 (strong stain). QuPath v0.2.0 was used for whole slide image analysis in some cases. The slides were scanned with Ventana iScan HT slide scanner (Roche, Basilea, Switzerland). The intensity threshold parameters for nuclear DAB detection and the intensity “Threshold” were 1+, 2+ and 3+, to 0.2, 0.4, and 0.6. The tissues were classified according to the H-score with these values.

### Statistical Analysis

The frequencies were expressed in percentage and a Chi-Square test was used to compare both cohorts of patients. The event time distributions for disease-free survival (DFS), PFS, and OS were estimated with Kaplan and Meier's method and compared using the log-rank statistic test, or the Cox proportional-hazards regression model. The variables shown by univariate analysis to be significantly associated with DFS, PFS, or OS were entered into a Cox proportional hazards regression model for multivariate analysis. The median values of methylation were used as cut-off values. The DFS was defined from the date of surgery to the date of the disease progression or death (of any cause). The PFS is defined as the time from the first day of therapy to the date of disease progression or death (of any cause). The OS measured from the first day of therapy in the case of borderline patients and the date of diagnosis for the resectable patients to the date of death. Statistical tests were performed with the IBM SPSS Statistics for Windows, Version 21 (Armonk, NY: IBM Corp). Graphical representations were created using GraphPad Prism version 8.0.2 (GraphPad Software, San Diego, California, USA, www.graphpad.com) with mean values and SD. The statistical analysis was performed with a non-parametric statistic test, using Kruskal-Wallis Test for independent samples with non-parametric distribution with Dunn's multiple comparison test as a multiple comparison test and Wilcoxon matched-pairs signed-rank test used as a two-sample paired non-parametric test.

## Results

### CHFR mRNA Expression Study Using the TCGA Database and Its Promoter Methylation Analysis by Illumina Probes

First, we analyzed the CHFR mRNA expression data using TCGA as a reference, due to the scarce number of publications regarding this approach in PDAC patients. The mRNA expression and clinical data were downloaded from the cBio Portal platform. We analyzed the data from 186 patients (TGCA, PanCancer Atlas), of whom 139/168 (82.7%) had PDAC. The Oncoprint summary shows only one *CHFR* missense mutation in D502G (0.7%) over these cases. On the other hand, we found other well-known classical mutations such as KRAS in 97/139 (69.8%), TP53 90/139 (64.7%), SMAD4 33/139 (23.7%), and CDKN2A 32/139 (23%). Focusing on CHFR, we observed a wide range of mRNA expressions in the same cohort (Supplementary Figure 1A). Significant differences in terms of PFS and OS were observed between decile subgroups, with better outcomes in those cases with higher levels of CHFR mRNA expression (Supplementary Figures 1B,C). Secondly, we downloaded all the probes of Illumina 450 K that overlap with the *CHFR* gene for patients but also a small cohort of healthy donors (*n* = 11) but did not find any significant differences in methylation patterns between these groups (Supplementary Figure 2).

### Clinical and Demographic Data of the Study Population

Thirty-five patients diagnosed with PDAC disease were enrolled in this study, with their respective samples analyzed. The median follow-up for the entire cohort was 15.44 months (4.76–49.25 months). The clinical and demographic data of patients included in our study are summarized in Table 1. Significant differences were found based on the pT stage (*p* = 0.006) and adjuvant treatment (*p* = 0.018) between both cohorts. When the entire cohort was analyzed, the pathological intra-vascular invasion was associated with significantly better PFS (6.92 vs. 2.85 months, *p* = 0.045). Similar results were observed for the same invasion pattern in terms of cancer-related death (27.07 vs. 11.33 months, *p* = 0.022), with this effect mainly assignable to the borderline cohort (27.07 vs. 10.57 months, *p* = 0.017). Significantly longer OS was detected in those of the patients that received systemic treatment after surgery (25.46 vs. 14.71 months, *p* = 0.05). In terms of postoperative mortality (<30 days after oncological surgery), no significant differences were observed between both groups (data not shown). In those of the patients treated with neoadjuvant chemotherapy, significant differences were perceived based on CA 19.9 levels at diagnosis (14.16 vs. 30.52 months, *p* = 0.005) and platelets-lymphocytes ratio (PLR) (15.27 vs. 30.52, *p* = 0.030). There was a non-significant trend toward improved DFS in those cases with absence of lymph node invasion in the resectable cohort (21.71 vs. 8.44 months, *p* = 0.080) or based on the pathological T stage (pT1-T2: 14.58 vs. pT3-pT4: 8.44 months, *p* = 0.24).

### CHFR Expression Analysis in FFPE Tissues Assessed by IHC

**Figure 1:**
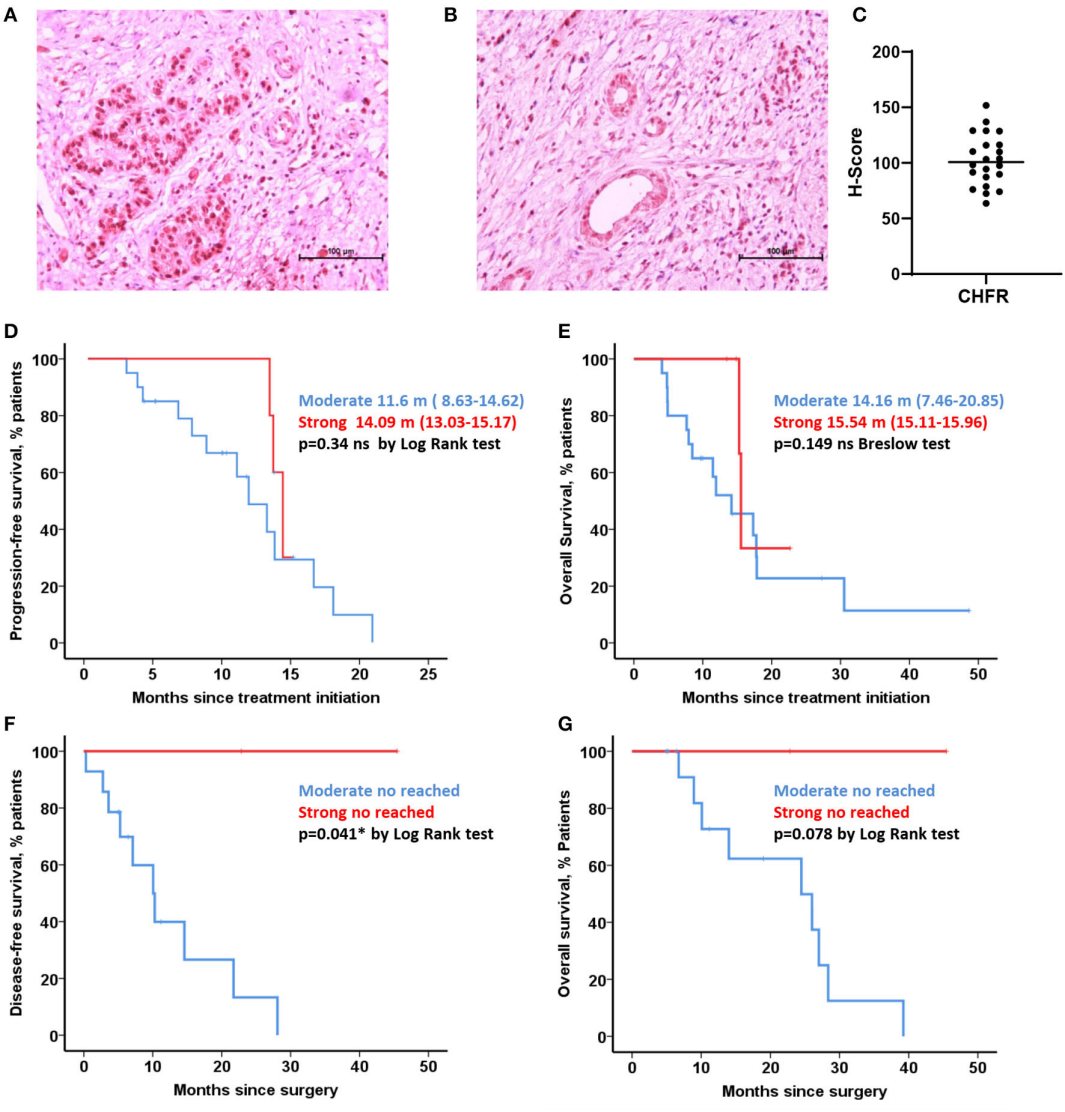
CHFR expression analysis. IHC pictures with differential expression, strong and weak stain **(A,B)** H-Score values from quantitative analysis performed in QuPath software with differential expression of CHFR **(C)**. Kaplan-Meier curves representing progression-free survival and overall survival for borderline cohort (15 patients with moderate stain vs. 4 strong stain) **(D,E)**. Kaplan-Meier curves representing disease-free survival and overall survival for resectable cohort (14 patients with moderate stain vs. 2 strong stain) (**F,G**, respectively). *Statistically significant differences (*p* < 0.05) and ns no significant differences (*p* > 0.05).

Checkpoint With forkhead and ring finger domains was expressed in all cases, regardless of whether the patients had received neoadjuvant treatment or not, with large differences in terms of intensity, although all cases were labeled as two or three (Figures 1A,B). CHFR was also expressed in all PDAC cell lines (Supplementary Figure 4). Further evaluation of IHC was done by an H-score through a quantitative analysis using the QuPath software in 22 randomly selected samples, which confirmed our initial results (Figure 1C). Statistically significant results were detected in the entire cohort in terms of PFS (12.74 months vs. not reached in the strong intensity cluster, *p* = 0.025). On the other hand, analysis of the borderline cohort did not reveal any significant difference with regard to the CHFR IHC score in terms of PFS or OS (Figures 1D,E). Similar to the entire group, significant differences for DFS were described between strong *vs*. moderate CHFR IHC intensity in the resectable cohort (Figure 1F). A trend for significance was also observed in terms of OS in the same cohort (Figure 1G).

### *CHFR* Methylation Analysis by MSP and Pyrosequencing in Borderline and Resectable Patients

**Figure 2:**
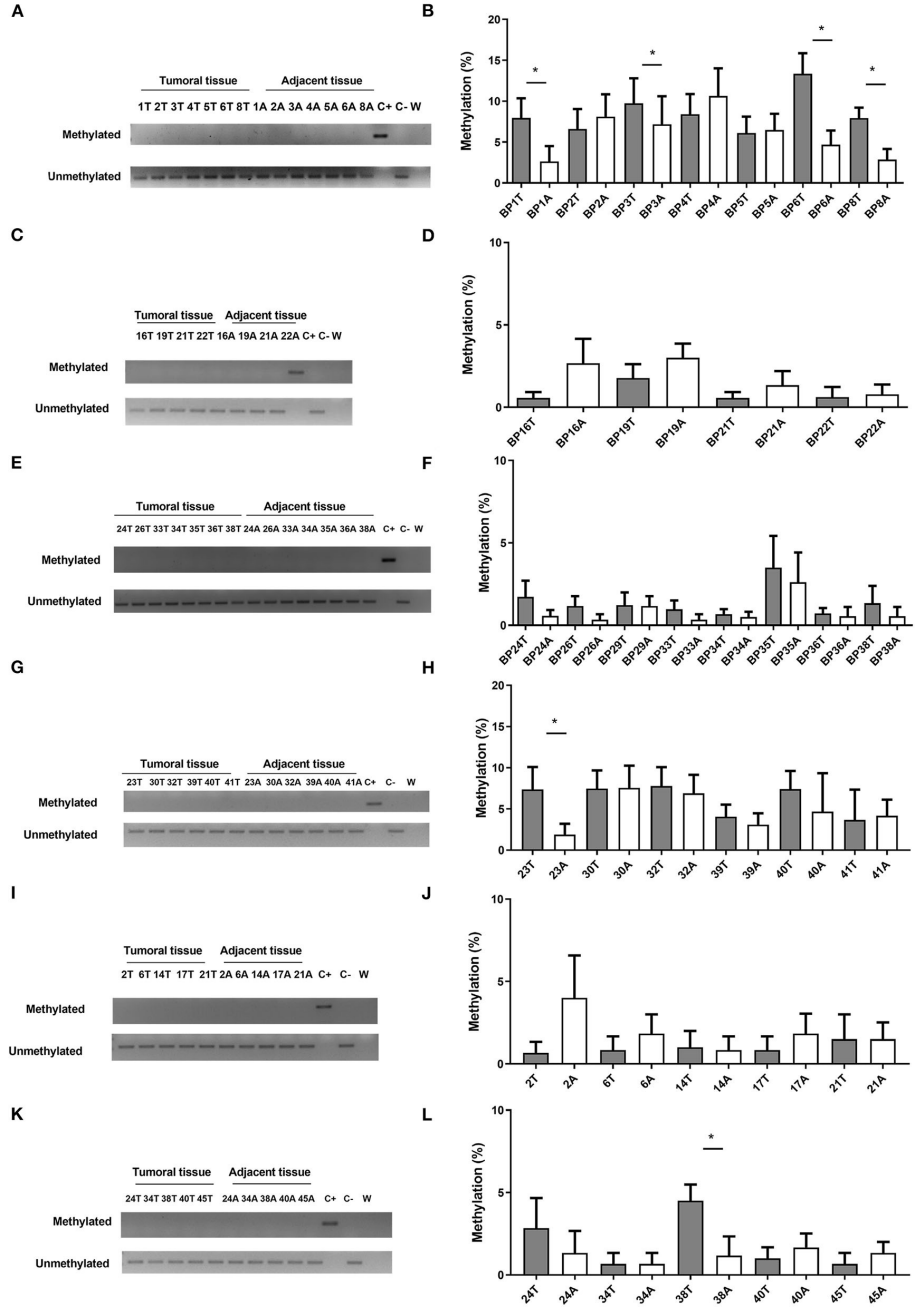
*CHFR* methylation analysis of human samples, borderline **(A–F)** and resectable cohort **(G–L)**. Methylation-Specific PCR (left) and Pyrosequencing Assay (right). T (tumoral tissue) and A (Adjacent tissue). *Statistically significant differences (*p* < 0.05). Columns represents mean and error bars correspond to standard deviations.

All cases established as borderline (Figures 2A–F) or resectable (Figures 2G–L), were classified as unmethylated, both in tumoral and non-tumoral adjacent tissue by MSP. Similarly, all PDAC cell lines were unmethylated (Supplementary Figure 4). Nevertheless, significant differences were observed between the tumoral tissue and adjacent tissue in four borderline cases by pyrosequencing. Uniformly, significant differences were found between the tumor and adjacent tissue in two cases in the initially resectable cohort by the same approach (*p* < 0.05).

During the pyrosequencing approach, there were no significant differences between both cohorts. Interestingly, CpG-4 presented a higher methylation percentage than the rest of CpG analyzed, with higher levels of methylation in this CpG associated with an absence of intravascular invasion (IV) in the resectable cohort (*p* = 0.041). Differently, higher levels of methylation of CpG-1 were significantly associated with IV in the borderline cohort (*p* = 0.023) (data not shown). Finally, significant differences were detected between tumor and adjacent tissue in the resectable cohort for CpC-6 (*p* = 0.031) (Supplementary Figure 5).

**Figure 3:**
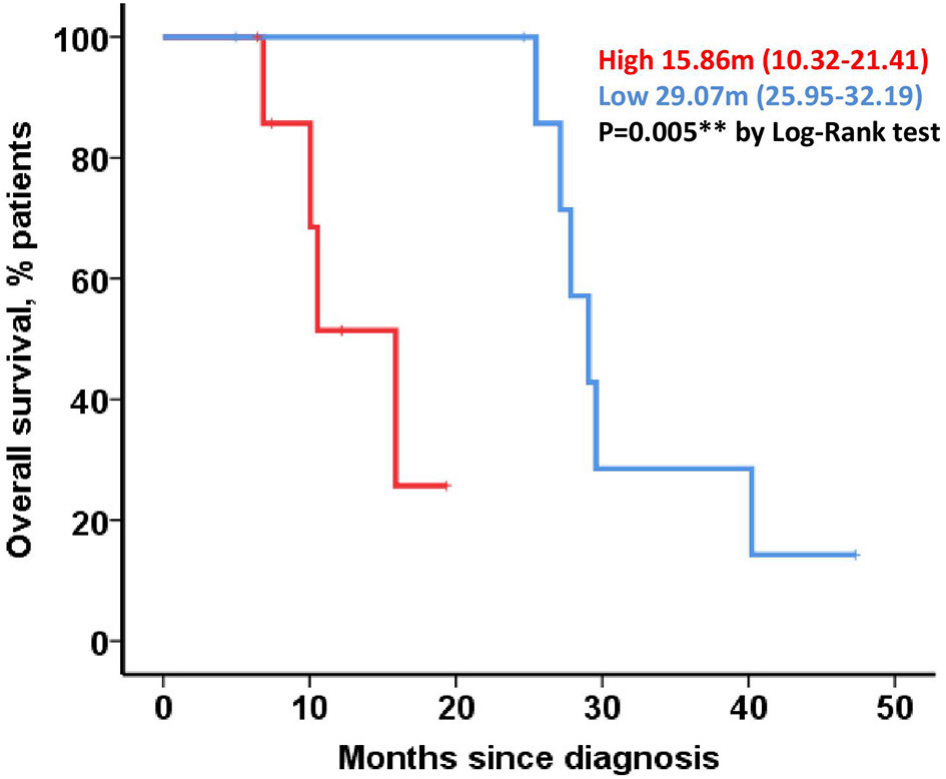
Correlation of *CHFR* methylation with clinical outcomes in resectable patients. Kaplan-meier curve representing OS for resectable cohort and dichotomized by CpG-2 median methylation percentage.

When the potential association of *CHFR* methylation levels with OS was analyzed in all the cohorts, lower levels of CpG-2 methylation were observed to be associated with longer OS in patients with primary tumor resected as the first therapeutic approach (29.07 vs. 15.86 months, *p* = 0.005) (Figure 3) and also in the entire cohort (27.13 vs. 15 months, *p* = 0.042).

### Multivariate Analysis

The vascular invasion (HR = 2.86; 95% CI: 0.16–1.55; *p* = 0.037) and intensity of CHFR by IHC (HR = 0.21; 95% CI: 0.05–0.95; *p* = 0.043) were established as independent prognosis factors of PFS when evaluating the entire cohort. Comparably, vascular invasion (HR = 3.50; 95% CI: 1.21–10.08; *p* = 0.021) and CpG-2 methylation (HR = 0.32; 95% CI: 0.105–0.96; *p* = 0.042) were independent prognosis factors for OS in the same group.

In those patients who received treatment before surgery, the CA 19.9 levels at diagnosis served as an independent prognostic factor for OS (HR = 0.101; 95% CI: 0.01–0.94; *p* = 0.044).

## Discussion

There is increasing evidence that CHFR plays a crucial role as a checkpoint in the cell cycle, regulating the entry into mitosis to prevent the proliferation of cells that have damage in their genome, and it has been proposed as a potential biomarker for several tumor types (7, 8). It is also widely known that, in contrast with other cell cycle checkpoints, *CHFR* is mostly found methylated in cancer (8). This previous evidence prompted us to gain a more precise understanding of the possible role of CHFR in PDAC, where there is very limited information. Firstly, through a TCGA data analysis, we found that CHFR mRNA has a wide range of expression without any relevant methylation pattern, suggesting that CHFR expression could be affected by post-transcriptional phenomena in PDAC. Secondly, our prospective study by IHC revealed that CHFR expression was observable in all samples, although with different intensity, and was associated with low methylation levels in most cases (~17% of methylation in both cohorts), which is in contrast with that described previously (35). This apparent difference could be related, at least in part, to the use of different IHC techniques and the selection of patients with neoadjuvant treatment in the retrospective study. Notwithstanding this, we found statistically significant differences based on IHC staining intensity, in the resectable and the entire cohort, in DFS and PFS, respectively. Additionally, CHFR IHC intensity was an independent prognosis factor for PFS in the entire cohort. Similarly, we found statistically significant differences in the OS based on the methylation profile described by pyrosequencing in both the resectable cohort and in the entire cohort. It was in the latter cohort where we were able to establish the utility of this methylation pattern as an independent prognostic for OS, similar to that which was previously described by the Human Protein Atlas (HPA) in kidney carcinoma (available at http://www.proteinatlas.org). Despite some of our initial results appearing somewhat contradictory, they are in fact supported by previous data (29) showing the prognostic role of *CHFR* promoter methylation in OS. To the best of our knowledge, this is the first study that shows the positive prognosis role in PFS of strong CHRF expression by IHC in patients with PDAC. This observation could be partially explained by the ambivalent role that CHFR seems to play in the process of carcinogenesis (29).

The use of PARP inhibitors, such as olaparib, as a maintenance treatment in advanced PDAC, remains controversial due to the lack of net benefit in most patients, in part explained by the lack of predictive biomarkers in this scenario (39). This implies a major problem in the development of clinical trials in this neoplasia (40) but also in the design and results of cancer clinical trials in general (41). In line with what has been recently described in gastric adenocarcinoma, linking the lack of CHFR expression and a higher sensitivity to PARP (26), it could be worthwhile to test the CHFR status in patients treated with this kind of drug family. Intriguingly, there are also different *in vitro* and *in vivo* approaches focusing on compounds with high inhibition properties of CHFR and PARP1 crosstalk, increasing synergy and effect of taxanes ordinarily used against this devastating disease (42).

Beyond the usual caveats associated with the limited number of cases enrolled in this kind of project, we recognize several limitations in our work. First, a bigger sample size would have potentially improved the present study by generating stronger findings in our uni- and multivariate analysis. This is the reason why we believe that it could still be necessary to carry out a new and more ambitious prospective study to validate our findings externally. Second, due to the possibility that neoadjuvant therapy may affect *CHFR* methylation patterns, we also enrolled patients who initially had their primary tumors removed, but did not notice any significant differences between the two groups. The fact that these two cohorts were treated differently may also explain the differences in the association between histopathological findings, such as intravascular spread and methylation patterns.

In conclusion, our results provide original evidence to suggest that the higher expression of CHFR and a specific methylation pattern over its promoter could likely help identify patients with better outcomes, among patients with PDAC who will receive surgery, regardless of when complementary treatment is administered. Undoubtedly, further research is required to elucidate the potential prognostic role of CHFR in patients diagnosed with PDAC in any stage, in addition to those who are treated with taxanes and/or PARP inhibitors.

## Data Availability Statement

The original contributions presented in the study are included in the article/Supplementary Material, further inquiries can be directed to the corresponding author/s.

## Ethics Statement

The studies involving human participants were reviewed and approved by Ethics Committee of the Government of Navarre. The patients/participants provided their written informed consent to participate in this study.

## Author Contributions

AV: conceptualization and writing review and editing. EA-P, JF-I, and JC: writing review and editing. IG-B: resources, writing the original draft, review, and editing. All authors listed have made a substantial, direct and intellectual contribution to the work, and approved it for publication.

## Funding

This work was funded by grants from the Department of Health from the Government of Navarra (Ref. 008-2018), REFBIO II Pyrenees Biomedical Network from Programa INTERREG V-A España-Francia-Andorra (Ref. BMK_PANC) and Sociedad Española de Oncología Médica (SEOM) to AV. IG-B was supported by a predoctoral fellowship from the Department of Economic Development Government of Navarre Ayudas para la contratación de doctorandos y doctorandas por empresas y organismos de investigación y difusión de conocimientos: doctorados industriales 2018–2020. Intensification Programme Navarrabiomed 2017-2021 Obra Social La Caixa Fundación Caja Navarra. This work has also been supported by the Spanish Ministry of Economy [MINECO; BFU2016-80360-R (to JC)] and the Ministry of Science and Innovation [MICINN; PID2019-105201RB-I00 (to JC)]. Instituto de Salud Carlos III, co-funded by European Union (ERDF/ESF, Investing in your future) [Predoctoral contract FI17/00282 (to EA-P)]. Junta de Andalucía (BIO-0139); GETNE2016 and GETNE2019 Research grants (to JC); and CIBERobn.

## Conflict of Interest

AV is employed by ICON plc. The remaining authors declare that the research was conducted in the absence of any commercial or financial relationships that could be construed as a potential conflict of interest.

## Publisher's Note

All claims expressed in this article are solely those of the authors and do not necessarily represent those of their affiliated organizations, or those of the publisher, the editors and the reviewers. Any product that may be evaluated in this article, or claim that may be made by its manufacturer, is not guaranteed or endorsed by the publisher.

## Supplementary Material

Supplementary Figure 1 Correlation of CHFR mRNA expression and clinical outcomes in TCGA cohort. **(A)** Scatter dot plot representing the mRNA expression levels of CHFR from Ilumina. Kaplan-Meier plots representing PFS and OS with decile groups by CHFR mRNA expression **(B,C)**. ^*^Statistically significant differences (*p* < 0.05). mRNA expression data was downloaded from mRNA expression, RSEM (Batch normalized from Illumina HiSeq_RNASeqV2) https://www.cbioportal.org/results/mRNA.

Supplementary Figure 2 Methylation analysis of *CHFR* gene using Ilumina probes. Upper graphs represent methylation analysis of *CHFR* gene in healthy donors **(A)** and PDAC patients **(B)**, methylation of *CHFR* gene with probes displayed in order of genomic order. Blue arrow, probe that correlate most with mRNA levels 13346603, without significance (Pearson = −0.21, Spearman = −0.38, *p*-value = 0.12). Green rectangle indicates promoter of *CHFR*. **(C)** *CHFR* gene representation by Genome browser. Methylation data was downloaded from www.firebrowse.org.

Supplementary Figure 3 CHFR methylation analysis in colorectal cell lines through Methylation specific PCR **(A)** and Pyrosequencing assay, columns represent mean and error bars correspond to standard deviations **(B)**. The analyzed cell lines were LOVO, HT29, HCT116, SW480, and DLD-1. HCT116 and DLD-1 were methylated, LOVO and HT29 hemi-methylated and SW480 unmethylated. CpG numbers are given by the order that were analyzed in pyrosequencing assay.

Supplementary Figure 4 CHFR methylation and expression analysis in pancreatic cancer cell lines, methylation specific PCR **(A)**, Pyrosequencing assay **(B)**, Western blot **(C,D)** and real time RT-PCR **(E)**. Columns represents mean and error bars correspond to standard deviations). ^*^,^**^ in the figures indicate significant (*p* < 0.05) and highly significant (*p* < 0.01) statistical differences, respectively.

Supplementary Figure 5 CHFR promoter methylation percentage for each CpG by pyrosequencing assay. Scatter dot plot graphs from **(A–F)** representing methylation percentage of a CpG comparing borderline and resectable cohorts, tumor and adjacent tissues, median with 95% CI. CpG numbers are given by the order that were analyzed in pyrosequencing assay. ^*^Statistically significant differences.

Supplementary Table 1 Primer design for CHFR promoter methylation analysis by MSP and pyrosequencing.

Supplementary Table 2 RT-PCR primer design.
