# Supplementary material for: Deciphering CHFR Role in Pancreatic Ductal Adenocarcinoma
Source: Front Med (Lausanne). 2021 Nov 19;8:720128. doi: 10.3389/fmed.2021.720128 (PMC8639583; doi:10.3389/fmed.2021.720128)
Supplement: Supplementary Figure 1 — Correlation of CHFR mRNA expression and clinical outcomes in TCGA cohort. (A) Scatter dot plot representing the mRNA expression levels of CHFR from Ilumina. Kaplan-Meier plots representing PFS and OS with decile groups by CHFR mRNA expression (B,C). *Statistically significant differences (p < 0.05). mRNA expression data was downloaded from mRNA expression, RSEM (Batch normalized from Illumina HiSeq_RNASeqV2) https://www.cbioportal.org/results/mRNA. [file Data_Sheet_1.pdf]

Supplementary Material

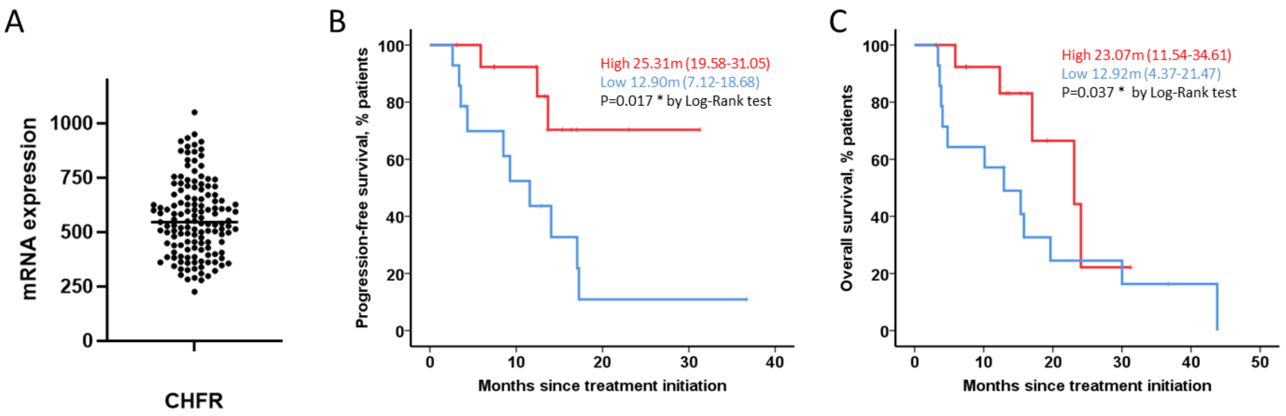

Supplementary Figure 1

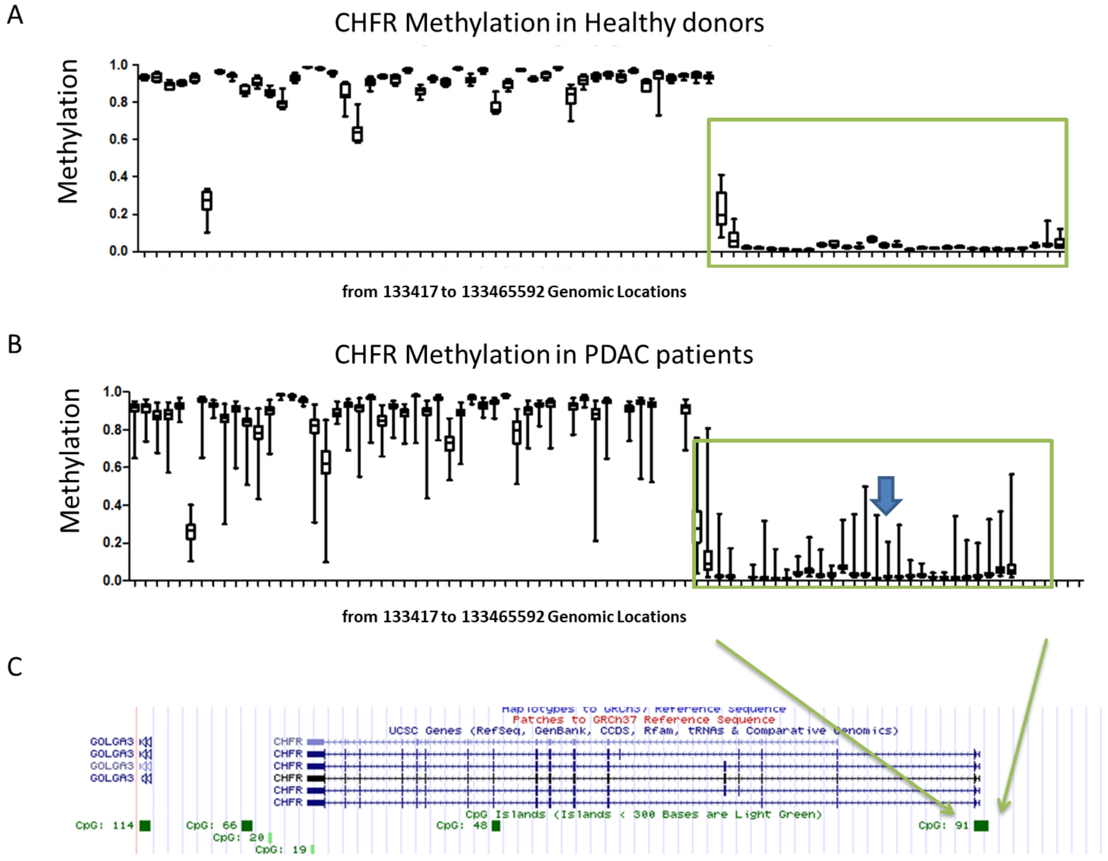

Supplementary Figure 2

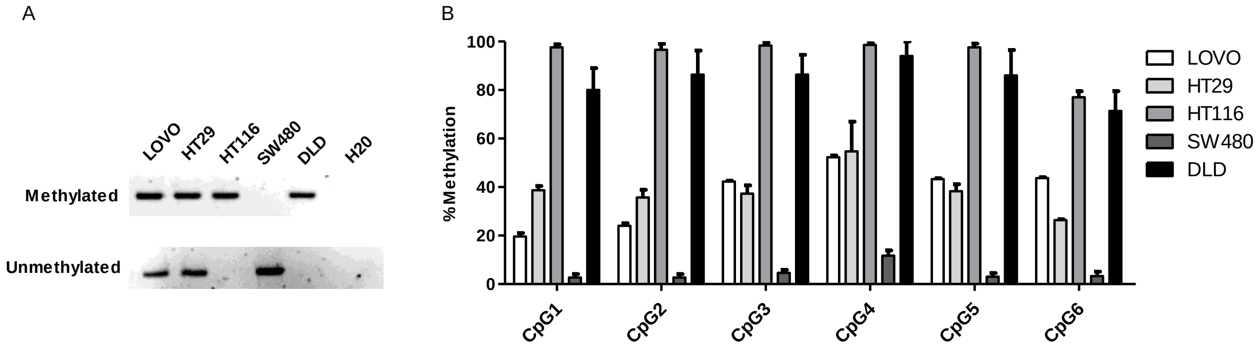

Supplementary Figure 3

**Methylation specific PCR**	**Primers**
**Methylation reaction**	
FW primer sequence	5'GTTATTTTCGTGATTCGTAGGCGAC-3'
RV primer sequence	5'-CGAAACCGAAAATAACCCGCG-3'
**Unmethylation reaction**	
FW primer sequence	5'-GATTGTAGTTATTTTTGTGATTTGTAGGTGAT-3'
RV primer sequence	5'-AACTAAAACAAAACCAAAAATAACCCACA-3'
**Pyrosequencing**	
Analysed sequence	ACATGGCGCCGACCGCAGCCACTTCCGTGATCCGCAGGCGA
PCR amplification primer FW	ATATGGYGTYGATYGTAGTTATTTTYGTGATTYGTAGGYGA
PCR amplification primer RV	TATCATGTCGATCGAGTCGTAGTATTCGTAGATCGTAGTCG

Supplementary Table 1

Gene		Sequence (5´-3´)	Template strand	Length	Tm	Product length
CHFR	FW1	GGCAGTGTCCTGAGTACAGA	Plus	20	60	136
	RV1	TAATCCTGGACTGCTGTCGTC	Minus	21	60	
	FW2	ATACCAGCACCAGTGGAACAG	Plus	21	60	133
	RV2	TAGAGGTATGCCACGTTGTGT	Minus	21	60	
GAPDH	FW	CAATGACCCCTTCATTGACC	Plus	20	60	106
	RV	GACAAGCTTCCCGTTCTCAG	Minus	20		

Supplementary Table 2

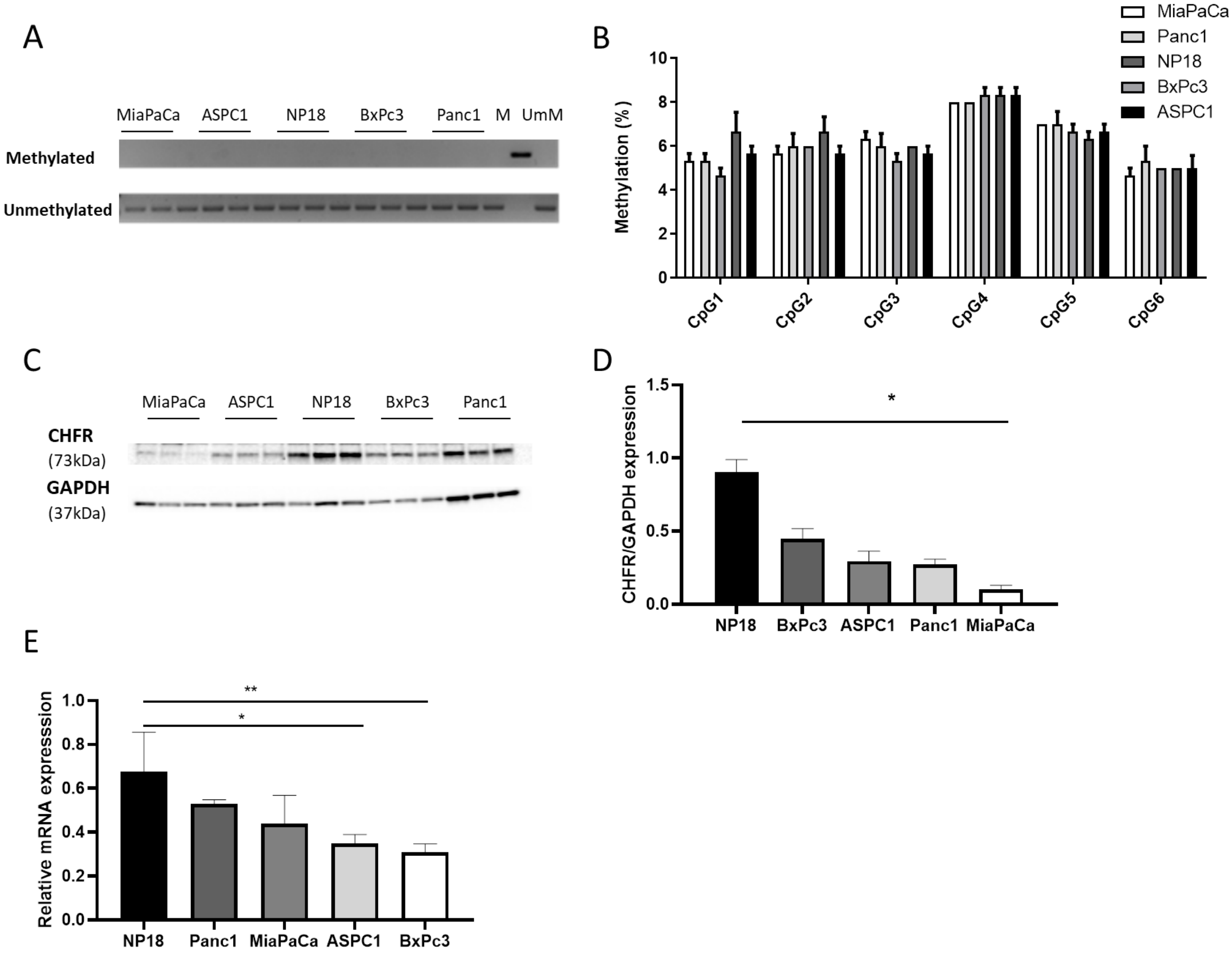

Supplementary Figure 4

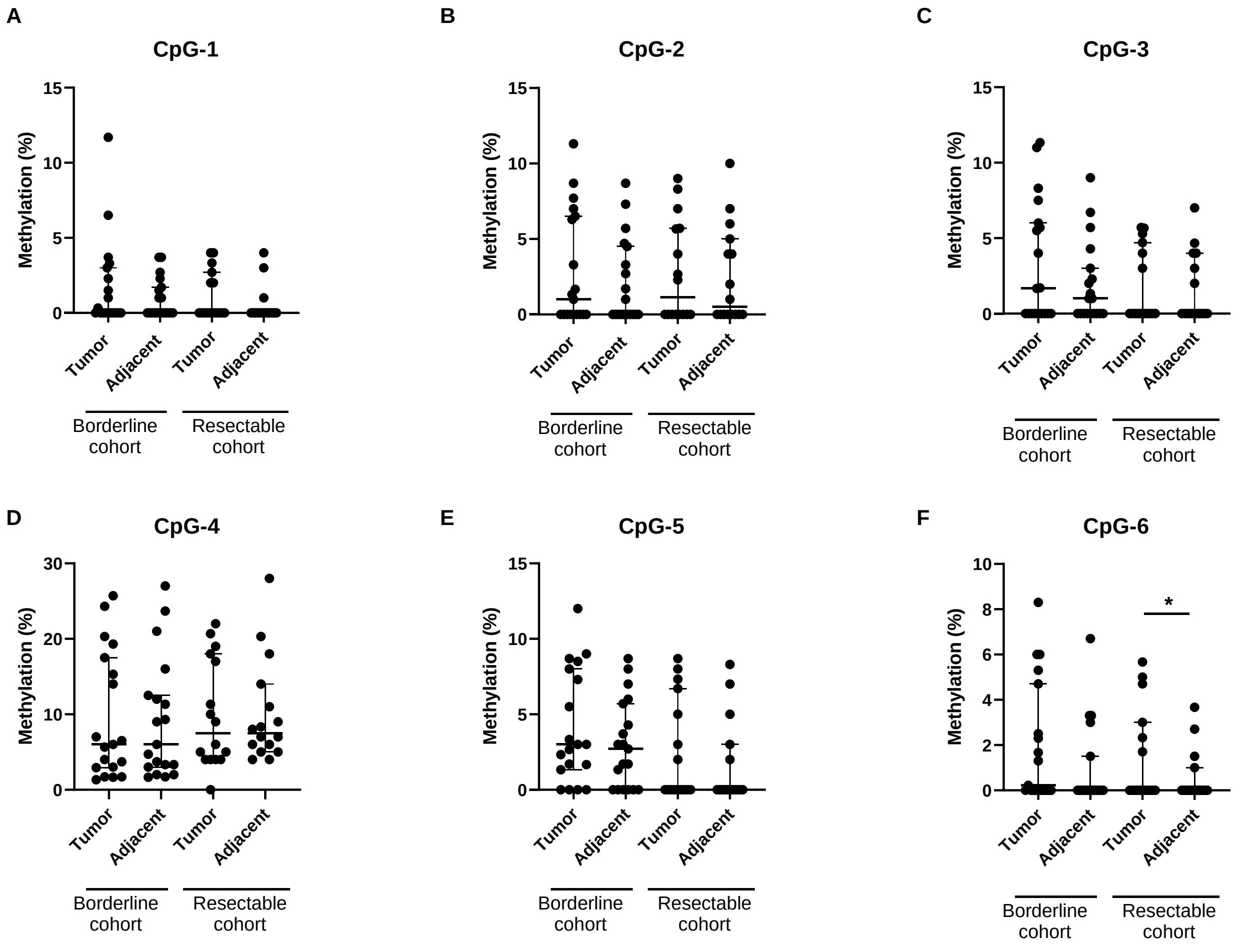

Supplementary Figure 5

Protocols

RNA extraction, reverse transcription and real time PCR

Cell pellets were re-suspended and mixed in 0.75–1ml Trizol reagent (15596018, Thermofisher) and incubated for 5min RT. Then, 0.2ml Chloroform (AM9730, Thermofisher) was added and tubes were shacked vigorously by hand for 15secs and incubated for 3min at RT. Samples were centrifuged 12000rpm 15min at 4°C. The upper waterphase was transferred into a new tube and 0.5ml of isopropanol was added to precipitate the RNA. Samples were incubated for 10min at RT and then centrifuged 12000rpm 10min at 4°C. RNA pellet was washed with 1ml 75% ethanol (in DEPC water) and mixed. Samples were centrifuged at 12000rpm for 5min at 4°C. Supernatant was removed and RNA pellet was re-suspended in 44µl DEPC water.

DNase treatment was performed in each sample 5µl 10× reaction buffer (AM2238 Invitrogen), 1µl RNase free DNase (40226, Invitrogen) were added and incubated for 15min at RT. Then, it was mixed with 5µl of STOP solution (5µl EDTA 25mM in RNA free water), heated for 10 min at 65°C and finally chilled on ice for 2min.

RT-PCR was carried out using random primers and SuperScript™ IV One-Step RT-PCR System (RR037A, Takara) according to the manufacturer's instructions. Briefly, cDNA was synthesized using 5µl of total RNA (500ng). 2µl 5× PrimeScript Buffer, 0.5µl PrimeScript RT Enzyme Mix I, 0.5µl Oligo dT Primer (50µM), 2µl Random 6 mers (100µM) were added to each sample and incubated 15min at 37°C and 5 seconds at 85°C in a thermal cycler.

qRT-PCR was performed with SYBR Green (4367659, Life technologies) following manufacturer's instructions. Briefly, 2µl (10ng/µl) of cDNA was mixed with 1µl H20 RNAase free, 5µl SYBR green, 1µl Forward (FW) primer and 1µl Reverse (RV) primer. See Supplementary Figures S2 and S3.

CHFR Western blot

Cellular pellets were obtained by cell scraping and then were lysed using lysis buffer (6M urea, 2M thiourea, 50mM DTT). Samples were incubated 30min in ice and then sonicated to aid in cell lysis and breakdown of DNA. Finally, samples were centrifuged at 20000g during 1h at 15ºC. The supernatant was kept in a new tube and the pellet was discarded. Protein concentration was performed using Bradford (Biorad). 18µg of protein per well was loaded into 4–15% CriterionTM TGX Stain-FreeTM (#5678085, BioRad, CA, USA) premade gels and resolved in an electrophoresis. Once the proteins were resolved in the gel, they were transferred to a nitrocellulose membrane using Trans-blot Turbo (Bio-Rad) at constant amperage of 2.5A and 25V of voltage for 7 minutes. The membranes were stained with Ponceau as control of transference. Membrane blocking was performed using 5% milk in TBS + 0.1% Tween during 1h. Primary antibody was used at a concentration of 1/500, WH0055743M1, Clone 1H3-A12 from Sigma. Horseradish peroxidase (HRP) - conjugated anti- mouse secondary antibody was used at 1:5000. Detection was performed using ECL- Enhanced Chemiluminescence (Perkin Elmer, MA, USA) and ChemiDocTM MP Imaging System (BioRad). To quantify protein bands, Image Lab v.5.2 (BioRad) software was used. Finally, protein quantifications were normalized using GAPDH as housekeeping gene (Supplementary Figures S3 and S4).
